# Advances in the Understanding of Reactive Oxygen Species-Dependent Regulation on Seed Dormancy, Germination, and Deterioration in Crops

**DOI:** 10.3389/fpls.2022.826809

**Published:** 2022-02-23

**Authors:** Wenjun Li, Yongzhi Niu, Yunye Zheng, Zhoufei Wang

**Affiliations:** ^1^The Laboratory of Seed Science and Technology, Guangdong Key Laboratory of Plant Molecular Breeding, Guangdong Laboratory of Lingnan Modern Agriculture, State Key Laboratory for Conservation and Utilization of Subtropical Agro-Bioresources, South China Agricultural University, Guangzhou, China; ^2^Yuxi Zhongyan Tobacco Seed Co., Ltd., Yuxi, China

**Keywords:** reactive oxygen species, seed germination, seed dormancy, seed deterioration, crops

## Abstract

Reactive oxygen species (ROS) play an essential role in the regulation of seed dormancy, germination, and deterioration in plants. The low level of ROS as signaling particles promotes dormancy release and triggers seed germination. Excessive ROS accumulation causes seed deterioration during seed storage. Maintaining ROS homeostasis plays a central role in the regulation of seed dormancy, germination, and deterioration in crops. This study highlights the current advances in the regulation of ROS homeostasis in dry and hydrated seeds of crops. The research progress in the crosstalk between ROS and hormones involved in the regulation of seed dormancy and germination in crops is mainly summarized. The current understandings of ROS-induced seed deterioration are reviewed. These understandings of ROS-dependent regulation on seed dormancy, germination, and deterioration contribute to the improvement of seed quality of crops in the future.

## Introduction

Reactive oxygen species (ROS) are known as a class of highly reactive and oxygen-bearing molecules including superoxide anion (O_2_^⋅–^), hydrogen peroxide (H_2_O_2_), hydroxyl radical (OH), and singlet oxygen (^1^O_2_) (Nathan and Ding, 2010). It has been well reported that ROS plays a pivotal function in the regulation of seed dormancy, germination, and deterioration (Kurek et al., 2019; Considine and Foyer, 2021). The low level of ROS as signaling particles promotes physiological dormancy release and triggers seed germination (Kumar et al., 2015; Considine and Foyer, 2021). However, the high level of ROS usually causes the orthodox seed deterioration under natural and artificial aging conditions by influencing lipid peroxidation, membrane permeability, defective proteins, antioxidant system, mitochondrial degradation, and DNA and RNA damages (Kurek et al., 2019). Therefore, keeping a balance in the ROS levels in seeds plays an important role in the regulation of seed dormancy, germination, and deterioration. In this study, the current advances in the regulation of ROS in seed dormancy, germination, and deterioration in crops are reviewed mainly considering three aspects: (1) the regulation of ROS homeostasis in seeds, (2) the crosstalk between ROS and hormones in seed dormancy and germination, (3) and ROS involving in seed deterioration.

## Regulation of Reactive Oxygen Species Homeostasis in Seeds

### Production of Reactive Oxygen Species in Seeds

**FIGURE 1:**
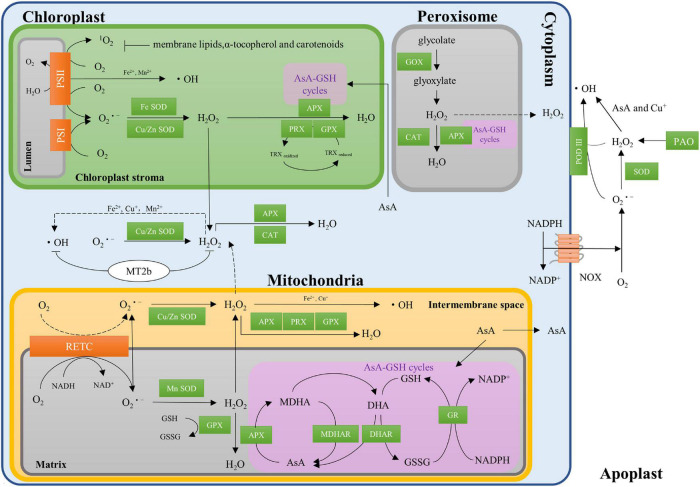
Regulation of reactive oxygen species (ROS) homeostasis in crop seeds. NADP^+^, nicotinamide adenine dinucleotide phosphate; NADPH, reduced form of NADP^+^; NAD^+^, nicotinamide adenine dinucleotide phosphate; NADH, nicotinamide adenine dinucleotide; NOXs, transmembrane NADPH oxidases; RETC, the respiratory electron transport chain; SOD, superoxide dismutase; GPX, glutathione peroxidase; AsA, ascorbate; GSH, glutathione; AsA-GSH cycles, the ascorbate-glutathione cycles; APX, ascorbate peroxidase; MDHAR, monodehydroascorbate reductase; DHAR, dehydroascorbate reductase; GR, glutathione reductase; MDHA, monodehydroascorbate; DHA, dehydroascorbate; GSSG, oxidized glutathione; PRX, peroxiredoxins; PSI/II, photosystem I/II; TRX, thioredoxin; MT2b, type 2 metallothioneins; CAT, catalase; GOX, glycolate oxidase; PAO, polyamine oxidase; POD III, secreted class III heme-containing peroxidases.

In dry seeds, the ROS are generated by the non-enzymatic reaction, mainly the autooxidation of lipids (Bewley et al., 2012). Lipids are easily oxidized as the main source of free radicals under low humidity conditions in seeds during dry storage, while a weakening lipid oxidation occurs with the increase in humidity (Singh et al., 2014). When seed imbibition with the water content increased from 8–10 to 50% or more, the production of ROS begins to switch from non-enzymatic system to the enzymatic system in seeds (Kibinza et al., 2006; Bazin et al., 2011; Basbouss-Serhal et al., 2016; Bailly, 2019). The mitochondrion is an important site for the main source of cellular ROS in seeds (Bailly, 2004). In the matrix of mitochondria, the oxygen consumed during electron transport is reduced to superoxide through the respiratory electron transport chain (RETC), and then the produced superoxide is converted to H_2_O_2_ by Mn superoxide dismutase (Mn-SOD) or Cu/Zn-SOD (Figure 1; Imlay, 2003; Møller et al., 2020). Chloroplasts are another vital source of ROS in photosynthesizing cells. Illumination of photosystem I (PSI) and photosystem II (PS II) generates O_2_^⋅–^, OH, and ^1^O_2_ (Pospíšil et al., 2004; Pospíšil, 2009; Richards et al., 2015), and the O_2_^⋅–^ is converted into H_2_O_2_ by Fe-SOD or Cu/Zn-SOD in chloroplasts (Waszczak et al., 2018). Meanwhile, the glycolates derived from chloroplast are converted into glyoxylate and H_2_O_2_ by glycolate oxidase (GOX) in peroxisomes (Considine and Foyer, 2021).

Transmembrane NADPH oxidases (NOXs) are well-studied cytosolic ROS-producing enzymes in plants (Ishibashi et al., 2015; Kai et al., 2016; Li et al., 2017). NOXs mediate the transfer of electrons from cytosolic NADPH, through flavin adenine dinucleotide (FAD) to penetrate the membrane, *via* hemes, to oxygen, leading to superoxide generation (Figure 1; Katerina and Cosa, 2016). The production of ROS also occurs in the apoplastic system (Richards et al., 2015; Waszczak et al., 2018). For example, H_2_O_2_ is generated by polyamine oxidase (PAO) during the catalytic synthesis between spermidine and spermine (Yoda et al., 2006; Moschou et al., 2008). The OH is converted from O_2_^⋅–^ and H_2_O_2_ by ascorbate (Fry, 1998; Schopfer, 2001) and secreted class III heme-containing peroxidases (POD III) in the apoplastic system (Mika et al., 2008; Heyno et al., 2011; Miura, 2012). Interestingly, OH could be produced from H_2_O_2_ through metal-based (Fe^2+^, Cu^+^, or Mn^2+^) Haber-Weiss or Fenton reactions in all mitochondria, chloroplasts, and cytoplasm (Pospíšil et al., 2004; Pospíšil, 2009). The enzymatic reactions in dry seeds are inactive, while the ROS generation by non-enzymatic reactions remains poorly understood in crops. It is important to understand the contributions of each site such as mitochondrion, peroxisomes, chloroplasts, cytoplasm, and apoplastic systems on ROS generation in the future.

### Scavenging System of Reactive Oxygen Species in Seeds

To keep ROS homeostasis in seeds, the internal antioxidant defense systems comprising of both enzymatic and non-enzymatic components are activated to relieve oxidative damages. Superoxide dismutase (SOD) including Mn-SOD, Fe-SOD, and Cu/Zn-SOD as important enzymatic components are widely distributed in the mitochondrial, chloroplasts, cytosol, and extracellular space of cells (Imlay, 2003). SODs can dismutate superoxide radicals into H_2_O_2_ (Figure 1). Then, H_2_O_2_ is converted into water and oxygen by catalase (CAT), glutathione peroxidase (GPX), peroxiredoxins (PRX), or by the ascorbate-glutathione (AsA-GSH) cycle (Figure 1). Several components such as ascorbate peroxidase (APX), monodehydroascorbate (MDHA), monodehydroascorbate reductase (MDHAR), dehydroascorbate reductase (DHAR), and glutathione reductase (GR) involve in the AsA-GSH cycle (Noctor and Foyer, 1998; Bailly, 2004), in which AsA is utilized as a specific electron donor to invert H_2_O_2_ to water by APX.

Several non-enzymatic components such as ascorbic acid (AsA, vitamin C), glutathione (GSH), hioredoxin (TRX), α-tocopherol (vitamin E), and carotenoids have been identified as the potent antioxidants in seeds (Figure 1). Of them, AsA and GSH have long been considered to function together in the AsA-GSH cycle (Foyer and Halliwell, 1976). Both GSH and TRX can be used as reducing substrates by GPX in the detoxification of H_2_O_2_ (Herbette et al., 2002). It has also been reported that membrane lipids, α-tocopherol (vitamin E), and carotenoids play important roles in clear ^1^O_2_ produced in the chloroplast (Krieger-Liszkay and Trebst, 2006; Ramel et al., 2012).

Metallothioneins (MTs) can bind metal ions through the thiol groups of their cysteine residues, which have been reported to be involved in the scavenging of ROS in the past decades. For example, the MTs can scavenge⋅OH and O_2_^⋅–^ in seeds (Figure 1; Hassinen et al., 2011), and overexpression of *OsMT2b* can reduce the H_2_O_2_ production in rice (Wong et al., 2004). Altogether, ROS homeostasis is controlled through a complex network of ROS production and scavenging systems, while its molecular mechanisms such as MTs involved in ROS homeostasis remain unclear. Maintaining ROS homeostasis plays a central role in seed dormancy, germination, and deterioration, and whether MTs involved in seed dormancy, germination, and deterioration needs further investigation.

## Reactive Oxygen Species Involved in Regulation of Seed Dormancy and Germination

### Roles of Reactive Oxygen Species in Regulation of Seed Dormancy and Germination

**FIGURE 2:**
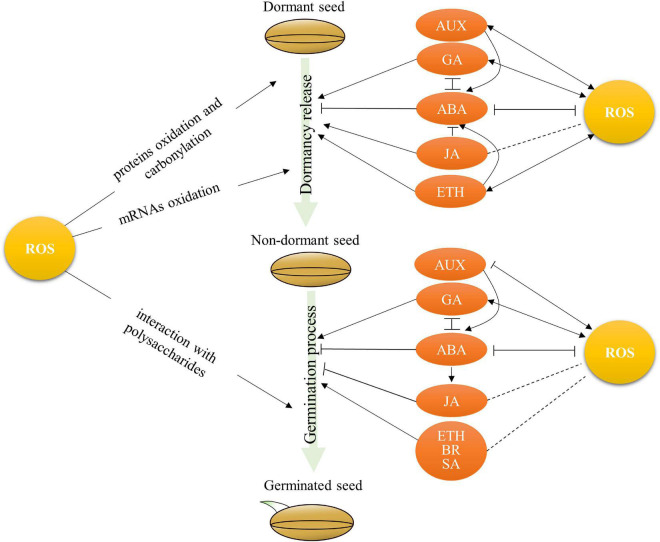
The crosstalk between ROS and hormones in regulation of seed dormancy and germination in crops. ABA, abscisic acid; GA, gibberellins; ETH, Ethylene; AUX, auxins; JA, jasmonates; SA, salicylic acid; BR, brassinosteroids. Arrows and lines with slanted dashes indicate positive and negative effects, respectively, and the dot lines indicate the putative effects.

The regulatory roles of ROS in dormancy release and seed germination in crops have been reported. For example, the non-enzymatic ROS generation frequently occurs in seeds contributing to dormancy release during desiccated seed storage (Finch-Savage and Leubner-Metzger, 2006). The accumulation of H_2_O_2_, OHs, and superoxide radicals has been widely observed during seed germination (Schopfer, 2001; Morohashi, 2002; Kranner et al., 2010; Li et al., 2017). Rice PAO OsPAO5 oxidizes PAs and releases H_2_O_2_, which is involved in coleorhiza-limited seed germination (Chen et al., 2016). The ROS produced by NOXs are involved in radical and root elongation during rice seed germination (Li et al., 2017). It has been reported that ROS-regulated dormancy release might be involved in mRNA oxidation (Bazin et al., 2011), protein carbonylation (Oracz et al., 2009), and oxidation (Bailly et al., 2008) in plants (Figure 2). For example, the oxidation of a specific subset of seed-stored mRNAs has been observed during dormancy alleviation by dry after-ripening. A total of 24 stored mRNAs, such as protein phosphatase 2C PPH1, mitogen-activated protein kinase phosphatase 1, and phenyl ammonia lyase 1, became highly oxidized during after-ripening in sunflower (Bazin et al., 2011). When seed germination, ROS can directly interact with polysaccharides of the cell wall that might promote cell elongation of the radical (Figure 2; Fry, 1998). A suitable ROS level will alleviate seed dormancy and trigger seed germination; however, the threshold of ROS level induced seed dormancy to germination, and its molecular mechanisms are understood poorly in most crops.

### Crosstalk Between Reactive Oxygen Species, Abscisic Acid, and Gibberellins in Regulation of Seed Dormancy and Germination

Abscisic acid (ABA) and gibberellins (GAs) are the main plant hormones that antagonistically mediate seed dormancy and germination (Finch-Savage and Leubner-Metzger, 2006). ABA induces seed dormancy and inhibits seed germination (Vaistij et al., 2013), while GA promotes dormancy release and facilitates seed germination (Gubler et al., 2008; Graeber et al., 2012). The involvement of ROS in seed dormancy and germination might be through the regulation of ABA and GA metabolisms in seeds (Figure 2). For instance, the biosynthesis of GA is stimulated by ROS through mitogen-activated protein kinase (MAPK) cascades (Kumar et al., 2015). The accumulation of H_2_O_2_ causes ABA degradation through influencing ABA catalytic enzyme (Ishibashi et al., 2015, 2017; Amooaghaie and Ahmadi, 2017; Li et al., 2018; Anand et al., 2019). The ascorbic acid and ROS involved in the inhibition of rice seed germination have been reported through influencing ABA levels (Ye et al., 2012). A recent study has shown that H_2_O_2_ enhances the germination capacity of primed tomato seeds due to the decrease in ABA/GA_3_ ratio by enhancing the expression of GA biosynthesis gene *GA3ox1* and ABA catabolism gene ABA 8-hydroxylase (ABA-H) (Anand et al., 2019). These reports illustrate that ROS plays a positive role in GA synthesis and ABA degradation, which, in turn, facilitates dormancy release and seed germination.

Interestingly, the accumulation of ROS affected by GA and ABA has been observed in seeds (Figure 2). Exogenous GA treatments have been elucidated to induce ROS production. For example, the content of H_2_O_2_ and O_2_^⋅–^ will be increased in caryopsis, embryo, and aleurone layer under GA_3_ treatment during the early imbibition stage in *Avena fatua* (Cembrowska-Lech et al., 2015). Similarly, exogenous GA_3_ and GA_4+7_ treatments could effectively promote the production of endogenous ROS during seed germination in *Brassica parachinensis* (Chen et al., 2021). However, the production of H_2_O_2_ and O_2_^⋅–^ is suppressed by ABA treatment in both dormant and non-dormant seeds in sunflower (El-Maarouf-Bouteau et al., 2015). Therefore, the balance of ROS and ABA/GA levels plays an important role in seed dormancy and germination. For example, the changing of balance between ABA and ROS is active in barley seed embryos after imbibition and then regulates seed dormancy and germination (Ishibashi et al., 2017). One major QTL *qSE3*, which encodes a K^+^ transporter gene *OsHAK21*, positively regulates seed germination and seedling establishment by increasing ABA biosynthesis and activating ABA signaling responses, and then decreasing H_2_O_2_ level in germinating seeds under salinity stress in rice (He et al., 2019). Furthermore, the antagonism between ABA and GA partially mediated by ROS during seed germination has also been observed in rice (Ye and Zhang, 2012).

The molecular mechanism of the relationship between ROS homeostasis and the ABA signaling pathway has been conducted in *Arabidopsis*. It showed that *Arabidopsis Abscisic Acid-Insensitive 5* (*ABI5*), a key component in ABA signaling, directly binds to the *CAT1* promoter and activates *CAT1* expression, and then ROS homeostasis is altered by *ABI5* though affecting *CATALASE* expression and catalase activity (Finkelstein, 1994a,b; Bi et al., 2017). *ABI4* directly combines with NADPH oxidase gene *RbohD* and *Vitamin C Defective 2* (*VTC2*), the key genes involved in the ROS production and scavenging, to modulate ROS metabolism during seed germination under salinity stress (Luo et al., 2021). However, the molecular mechanisms of the crosstalk between ROS, ABA, and GA in the regulation of seed dormancy and germination are still poorly understood in crops.

### Crosstalk Between Reactive Oxygen Species and Other Hormones in Regulation of Seed Dormancy and Germination

Other hormones such as ethylene (ETH), auxins, and jasmonates (JA), salicylic acid (SA), and brassinosteroids (BR) are also involved in the regulation of seed dormancy and germination (Figure 2). Several reports indicate that ROS might be also involved in the regulation of ETH and auxins on seed dormancy or germination in crops. For instance, the treatment of ROS-generated compound methylviologen increases the expression of ETH receptors *ETR2* and ETH-responsive factors *ERF1* in dormant sunflower embryos (Oracz et al., 2009). Exogenous ETH promotes dormancy release due to the ROS accumulation in dormant embryonic axes through activating NADPH oxidase by ETH in sunflower (El-Maarouf-Bouteau et al., 2015). The interaction of ROS, ABA, and ETH has been reported to regulate dormancy release in sunflowers (El-Maarouf-Bouteau et al., 2015). However, whether the crosstalk between ROS and ETH is involved in the regulation of seed germination remains unclear in crops. Biochemical analysis has revealed that the increase in H_2_O_2_ and the activation of peroxidases promote the oxidative degradation of IAA (Gazarian et al., 1998). In *Arabidopsis*, auxin promotes the production of superoxides such as NADPH oxidase and superoxide oxidase, while reducing the expression of antioxidant enzymes such as catalase and ascorbate oxidase (Iglesias et al., 2010; KrishnaMurthy and Rathinasabapathi, 2013). Similarly, the exogenous auxin regulates H_2_O_2_ metabolism by affecting the expression and activity of CuZn-superoxide dismutase, catalase, and peroxidase in tomatoes (Ja et al., 2009). The inhibition of auxin-stimulated NADH oxidase activity has been reported in the elongation growth of soybean hypocotyls (Morre et al., 1995). The crosstalk between ROS and auxin in the regulation of seed dormancy or germination might be through the influencing ROS homeostasis and auxin level.

It has been well reported that JA control seed dormancy and germination mainly through modulating ABA metabolism or signaling pathway. For example, JA promotes dormancy release through the suppression of ABA biosynthesis *Ta9-cis-EPOXYCAROTENOID DIOXY-GENASE TaNCED1* and *TaNCED2* in wheat (Xu et al., 2016), while ABA promotes JA biosynthesis to synergistically inhibit seed germination in rice (Wang et al., 2020). However, whether the crosstalk between ROS and JA is involved in the regulation of seed dormancy and seed germination needs further investigation in crops (Figure 2). Meanwhile, it has been reported that SA promotes seed germination under high salinity by modulating antioxidant activity in *Arabidopsis* (Lee et al., 2010). Spatiotemporal variations in SA and H_2_O_2_ have been observed in sunflower seeds during the transition from dormancy to germination (Vigliocco et al., 2020). Exogenous BRs increase seed germination under stress conditions in *Brassica juncea* (Soares et al., 2020). However, whether the crosstalk between ROS, SA, and BR is involved in the regulation of seed dormancy and germination needs further investigation in crops (Figure 2). It is an intriguing task to tackle the complicated network between ROS and hormones in regulating seed dormancy and germination in crops.

## Reactive Oxygen Species Involved in Regulation of Seed Deterioration

### Seed Deterioration and Reactive Oxygen Species Accumulation

Seed viability will be undermined due to deterioration under natural and artificial aging conditions in crops. For example, seed germination will be remarkably reduced after 18- and 24-months seed storage in sunflower in the dry airtight container under ambient temperature (Huang et al., 2021). In sweet corn, seed germination will be significantly decreased after 30 days of natural aging (17–28°C, 30–60% of RH) or after 0.5 h of artificial aging treatment (45°C, 100% of RH) (Zhang et al., 2022). Similarly, accelerated seed deterioration has been observed in rice and common bean under elevated temperature (45°C) and moisture (100% of RH) conditions (Dantas et al., 2019). When the seed experiences deterioration, the deleterious ROS will be largely accumulated in the seeds. In orthodox seeds, seed deterioration is caused by the reduction of antioxidant enzymes and the high accumulation of ROS (Ebone et al., 2019). Similarly, the higher accumulation of H_2_O_2_ could aggravate desiccation damage of recalcitrant seeds such as tea (*Camellia sinensis*) under chilling or drying stress (Chen et al., 2011). Thus, the instability of the intracellular ROS status causes the consequent oxidative damages to reduce seed viability in plants (Sano et al., 2016; Ebone et al., 2019).

### Mechanisms of Reactive Oxygen Species in Regulation of Seed Deterioration

**FIGURE 3:**
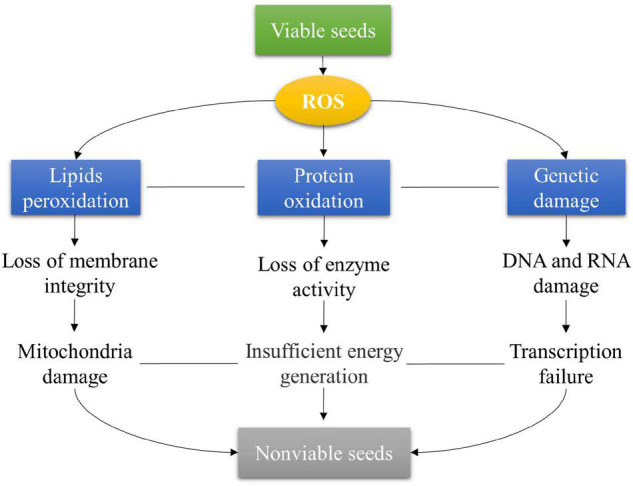
Seed deterioration caused by ROS under natural and artificial aging conditions in crops.

Seed deterioration caused by ROS is mainly involved in lipid peroxidation, protein oxidation, DNA and RNA damages, and repair system damage (Figure 3). Lipid peroxidation is the primary factor influencing seed deterioration (Zhao et al., 2021). The excessive ROS attack the membrane polysaturated fatty acids and divide the long-chain fatty acids into small compounds, which affects membrane permeability and ionic homeostasis (Oenel et al., 2017; Ebone et al., 2019). Lipid peroxidation disrupts many organelles, especially the mitochondrial damage influencing energy production for seed germination. Moreover, the end products formed from lipid peroxidation are also involved in seed deterioration. For example, the malondialdehyde (MDA) level is widely regarded as an indicator of lipid peroxidation and oxidative stress in seeds (Marnett, 1999). The 4-hydroxy-2,3-non-enal (HNE) influences the expression of genes by reacting with nucleic acids, proteins, and phospholipids during seed deterioration (Oenel et al., 2017).

Excessive ROS induces protein oxidation that is associated with seed deterioration in many crops (Figure 3). For instance, increased protein carbonylation has been observed in aged lettuce seeds (Adetunji et al., 2021) and *Vigna unguiculata* seeds (Boucelha et al., 2021). Several amino acids such as arginine, lysine, proline, and threonine residues with nucleophilic centers react with the reactive carbonyl species (RCS) derived from HNE and MDA (Smakowska et al., 2014; Satour et al., 2018; Biswas et al., 2020). However, *Arabidopsis NADP-ME1* catalyzes the oxidative decarboxylation of malate to pyruvate that protects protein oxidation, especially carbonylation during seed deterioration (Yazdanpanah et al., 2019). The accumulation of carbonylated proteins results in the disruption of the tricarboxylic acid (TCA) cycle, electron transport chain (ETC) enzymes, and glycolysis in seeds (Yin et al., 2017; Chen et al., 2019; Zhang et al., 2021).

The disruption of DNA and RNA induced by ROS is also involved in seed deterioration (Figure 3; Sano et al., 2016). The nucleotide damage usually occurs in the hydroxylation at the C-8 position in guanine (G) to form 8-oxoguanine (8-oxoG) during seed deterioration, which results in transversion mutations (GC → TA) due to a mispair of 8-oxoG with adenine (A) or cytosine (C) during DNA replication (Johnston et al., 2010; Boesch et al., 2011; Sano et al., 2016; Ebone et al., 2019). The degradation of RNA is also observed in seed deterioration during storage in soybean (Fleming et al., 2017, 2018). A significant reduction in mean RNA integrity number (RIN) has been observed in soybean seeds after being stored dry at 5°C for 1–27 years, which is positively associated with seed germination (Fleming et al., 2017). The fragmented mRNA in dry-stored soybean seeds leads to inefficient translation and faulty proteins and then results in the loss of germination capacity (Fleming et al., 2018). To avoid seed deterioration, the repair system is induced during seed germination (Long et al., 2015). For example, the DNA and protein damages can be repaired by base excision repair (BER) (Sano et al., 2016) and L-isoaspartyl methyltransferase (PIMT), respectively (Mudgett et al., 1997; Bewley et al., 2012; Sano et al., 2016). Nevertheless, if the extent of seed deterioration is beyond the ability of the repair system, the loss of seed vigor will not be restored. Overall, the molecular mechanisms of seed deterioration are still poorly understood in crops.

## Conclusion

In conclusion, ROS is mainly produced by lipid oxidation in dry seeds and enzymatic catalysis in hydrated seeds, respectively. The processes of ROS production occur in the mitochondrion, peroxisomes, chloroplasts, cytoplasm, and apoplastic systems in seeds. The antioxidant systems include the enzymatic and non-enzymatic systems involved in the scavenging ROS in seeds. Maintaining ROS homeostasis plays a central role in seed dormancy, germination, and deterioration in crops. The crosstalk between ROS, ABA, and GA in the regulation of seed dormancy and germination has been well investigated. However, the crosstalk between ROS and other hormones such as ETH, JA, SA, and BR involved in the regulation of seed dormancy and seed germination remains unclear in crops. The seed deterioration caused by excessive ROS accumulation is widely considered due to influencing lipid peroxidation, protein oxidation, DNA and RNA damages, and repair system damage in seeds under natural and artificial aging conditions in crops. Overall, the mechanisms of ROS regulation on seed dormancy, germination, and deterioration remain poorly understood in crops.

## Author Contributions

ZW and YZ designed the manuscript. ZW, WL, YZ, and YN wrote the manuscript. All authors contributed to the article and approved the submitted version.

## Conflict of Interest

YN and YZ were employed in Yuxi Zhongyan Tobacco Seed Co., Ltd. The remaining authors declare that the research was conducted in the absence of any commercial or financial relationships that could be construed as a potential conflict of interest.

## Publisher’s Note

All claims expressed in this article are solely those of the authors and do not necessarily represent those of their affiliated organizations, or those of the publisher, the editors and the reviewers. Any product that may be evaluated in this article, or claim that may be made by its manufacturer, is not guaranteed or endorsed by the publisher.
